# Reduced levels of nitrated α-synuclein in the protective effect of harpagoside on rotenone-induced cellular models of Parkinson’s disease

**DOI:** 10.3389/fcell.2025.1624315

**Published:** 2025-11-10

**Authors:** Juan Lang, Zhongkui Xiong

**Affiliations:** 1 Department of Pathology, Shaoxing People’s Hospital, Shaoxing, Zhejiang, China; 2 Department of Radiation Oncology, Shaoxing Second Hospital, Shaoxing, Zhejiang, China; 3 Department of Medical Imaging, School of Medicine, Shaoxing University, Shaoxing, Zhejiang, China

**Keywords:** iridoid, harpagoside, Parkinson’s disease (PD), α-synuclein, nitric oxide, nitration, rotenone

## Abstract

**Introduction:**

Parkinson’s disease (PD), ranking as the second most common neurodegenerative disorders following Alzheimer’s disease, involves the progressive accumulation of misfolded proteins in affected neural tissues. This pathological process appears linked to overproduction of reactive oxygen species (ROS) and reactive nitrogen species (RNS). Significantly, more than 30% of proteins aggregated in Lewy bodies exhibit post-translational modifications, particularly RNS-mediated nitration and S-nitrosylation. Experimental evidence suggests that α-synuclein nitration promotes its misfolding and neurotoxic effects in PD models.

**Methods:**

To model PD pathology, rotenone was applied to induce cellular damage in Neuro-2A (N2A) cells and BV-2 microglial cells. Three iridoid constituents from *Scrophularia ningpoensis Hemsl*, harpagoside, acetylharpagide, and haragide, were investigated for their neuroprotective potential against rotenone-induced cytotoxicity, with catalpol serving as reference compound. Cell viability was assessed using the CCK-8 assay, nitric oxide (NO) levels were measured via the nitroso assay, nitric oxide synthase (NOS) activity was determined by enzyme-linked immunosorbent assay (ELISA), and nitrated α-synuclein expression was evaluated through immunocytochemistry.

**Results:**

Our studies revealed that both acetylharpagide and harpagoside demonstrated substantial cytoprotective effects on rotenone-treated N2A cells. Further investigation focusing on harpagoside showed its ability to suppress NO generation and inhibit α-synuclein nitration.

**Discussion:**

Detailed pathway analysis indicated that harpagoside’s protective actions involved regulation of the nuclear factor-κB (NF-κB)/NOS/NO/α-synuclein nitration signaling cascade.

## 1 Introduction

Parkinson’s disease (PD) ranks as the second most common neurodegenerative condition globally, trailing only Alzheimer’s disease in prevalence. In populations aged over 60 years, PD affects roughly 1% of individuals according to epidemiological data (Lee and Gilbert, 2016). This disorder manifests the gradual deterioration of dopaminergic neurons within the substantia nigra (SN) region of the brain (Ding et al., 2023). Characteristic motor dysfunction emerges as a primary clinical feature, becoming clinically detectable only when neuronal loss in the SN reaches 40%–80% (Lorente-Picon and Laguna, 2021; Miller and O'Callaghan, 2015). Pathologically, PD demonstrates abnormal accumulation of misfolded α-synuclein (α-Syn) proteins that form distinctive intracellular deposits called Lewy bodies (LBs) (Mahul-Mellier et al., 2020; Wakabayashi et al., 2013). These pathological structures exhibit dense clusters of cellular organelles combined with multilayered lipid membranes (Shahmoradian et al., 2019). The aggregation of malformed proteins represents a common pathological mechanism across neurodegenerative disorders, potentially driving synaptic dysfunction and neuronal degeneration. Recent studies suggest these protein abnormalities may arise from excessive generation of reactive oxygen species (ROS) and reactive nitrogen species (RNS) within affected neural tissues (Nakamura et al., 2021). Notably, approximately one-third of LB-associated proteins undergo specific post-translational modifications, particularly nitration and S-nitrosylation processes mediated by RNS (Stykel and Ryan, 2022; Kim et al., 2014).

Toxic mechanisms mediated by nitric oxide (NO) involve a greater array of reactive species and post-translational modifications (Laranjinha et al., 2021). Nitration of tyrosine residues and covalent α-Syn dimer formation are notable modifications found in post-mortem brain sections from PD patients (Kumar et al., 2014). Experimental models demonstrate that nitration of α-Syn facilitates protein misfolding and enhances neurotoxic effects in PD pathogenesis (Stykel and Ryan, 2022). Synthetic alpha-synuclein fibrils cause mitochondrial dysfunction and selectively promote dopamine neurodegeneration, partly through nitric oxide (NO) production mediated by inducible nitric oxide synthase (iNOS) (Tapias et al., 2017). Persistent neuroinflammation leads to chronic degeneration of the nigrostriatal dopamine pathway, causing α-Syn accumulation and LB-like inclusions in nigral neurons. Inhibitors of iNOS in activated microglia effectively block both α-Syn pathology and neurodegeneration (Gao et al., 2011). In a mouse model of PD induced by the environmental toxin 1-methyl-4-phenyl-1,2,3,6-tetrahydropyridine (MPTP), increased nitration of α-Syn was demonstrated (Hou et al., 2017). The overexpression of iNOS increased NO production, leading to the nitration and misfolding of α-Syn in neurons (Stone et al., 2012). Long-term activation of microglia and astrocytes is essential in PD progression, involving the NF-κB signaling pathway that leads to excessive NO (Carbone et al., 2009). Inhibiting iNOS expression in astrocytes triggered by MPTP protects striatal neurons (Carbone et al., 2009). NO release in LPS-stimulated BV2 microglia is likely mediated by the protein kinase B (PKB, Akt)/NF-κB signaling pathway (Ni et al., 2025). Modulation of NO levels has been observed in both central nervous system neurons and glial cells, such as microglia and astrocytes, which are activated during neuroinflammatory responses linked to PD. This modulation may offer potential benefits for the use of non-steroidal anti-inflammatory drugs (Doherty, 2011).

Contemporary therapeutic approaches continue to focus on symptom management rather than effectively preventing disease development or arresting pathological advancement during initial stages (Lorente-Picon and Laguna, 2021). A critical obstacle in formulating effective treatments lies in the substantial degeneration of dopaminergic neurons that typically precedes clinical detection, making these cells minimally responsive to treatment modalities by diagnostic confirmation (Koeglsperger et al., 2023). *Scrophularia ningpoensis Hemsl.*, employed in Asian traditional medicine for over two millennia, demonstrates therapeutic potential through its extract of roots (*Scrophulariae Radix*), which has been documented in historical texts for managing inflammatory conditions and other disorders (Lee et al., 2021; Ren et al., 2021). Similarly, *Harpagophytum procumbens* has served as a therapeutic agent for diverse medical purposes across multiple generations, with its medicinal applications extensively documented in ethnopharmacological records (Menghini et al., 2019). Phytochemical investigations of *Scrophularia scorodonia* roots have led to the isolation and identification of harpagoside, 8-O-acetylharpagide, and harpagide (de Sa et al., 2000). As the principal bioactive constituent, harpagoside serves as a key marker for assessing the pharmacological quality of *S. ningpoensis Hemsl.* and *Harpagophytum procumbens* (Menghini et al., 2019; Ferrante et al., 2017). Clinical studies highlight its dual therapeutic potential as both an anti-inflammatory agent and analgesic (Gxaba and Manganyi, 2022; Fu et al., 2024). The antioxidant and anti-inflammatory effects of harpagoside have been documented. Experimental models demonstrate its efficacy in alleviating memory deficits associated with chronic cerebral hypoperfusion through modulation of phosphatase and tensin homolog deleted on chromosome ten (PTEN) gene activity (Chen et al., 2018). *In vitro* studies using rat cortical cell cultures revealed E-harpagoside’s neuroprotective properties against glutamate-induced toxicity (Kim et al., 2002). Both stereoisomers (E and Z forms) exhibited cognition-enhancing effects via acetylcholinesterase inhibition and antioxidant pathways (J et al., 2008). Harpagoside protects against hypoxia-induced toxicity in microglial cells via the hypoxia-inducible factor-1α (HIF-1α) pathway (Sheu et al., 2015). Harpagoside inhibits the expression of iNOS and cyclooxygenase-2 (COX-2) induced by lipopolysaccharide through the suppression of NF-κB activation, as demonstrated in human HepG2 hepatocarcinoma and RAW 264.7 macrophage cell lines (Huang et al., 2006).

Earlier investigations have established that harpagoside exerts neuroprotective properties in both *in vivo* and *in vitro* PD models through glial cell line-derived neurotrophic factor (GDNF) upregulation and mitochondrial function preservation (Lang and Xiong, 2025; Sun et al., 2012). Nevertheless, the potential role of nitrated α-Syn modification in mediating harpagoside’s therapeutic effects remains unexplored. This investigation seeks to elucidate the compound’s regulatory impact on the NF-κB/NOS/NO/α-Syn nitration signaling cascade within rotenone-induced cellular PD models.

## 2 Materials and methods

### 2.1 Cell lines

The murine BV-2 microglial cell line was procured from Shanghai Fuxiang Biotechnology Co., Ltd., China whereas the Neuro-2A (N2A) cell line originated from the Cell Bank of the Typical Culture Preservation Committee at the Chinese Academy of Sciences.

### 2.2 Chemicals and reagents

**FIGURE 1:**
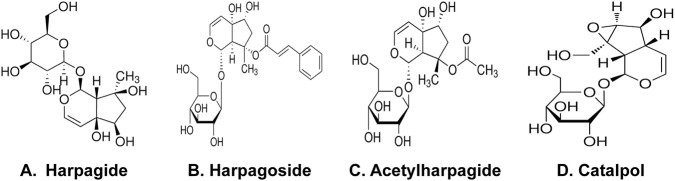
Harpagide and harpagoside **(A,B)** were supplied by Chengdu Push Biotechnology Co., Ltd (China). Acetylharpagide and catalpol **(C,D)** came from the China National Institute of Food and Drug Control.

Harpagide and harpagoside (Figures 1A,B) were supplied by Chengdu Push Biotechnology Co., Ltd. (China). Acetylharpagide and catalpol (Figures 1C,D) came from the China National Institute of Food and Drug Control. Sigma–Aldrich provided rotenone and dibutyryl-cyclic adenosine monophosphate (db-cAMP). Cell culture reagents including Dulbecco’s modified Eagle medium (DMEM), minimal essential medium (MEM), fetal bovine serum (FBS), and trypsin were sourced from Invitrogen. Primary mouse monoclonal antibodies targeting human α-Syn (nitrated Tyr 125 and Tyr 133) (24.8) (Cat No: NBP1-26380) (host: mouse, species: human, mouse) originated from Novus Biologicals. Wuhan Boster Biological Engineering Co., Ltd. (China) supplied secondary antibodies (Mouse/rabbit IgG immunohistochemistry kit SA1020, Cat No: 08E25C), streptavidin-biotin complex (SABC), and diaminobenzidine (DAB). The NO detection kit was purchased from Abcam, while the iNOS enzyme-linked immunosorbent assay (ELISA) kit was acquired from EIAab Science Inc., Wuhan, China. Cell viability was assessed using CCK-8 from Dojindo Chemistry Research Institute (Japan), and protein quantification employed the bicinchoninic acid (BCA) assay kit from Thermo Scientific.

#### 2.2.1 Culture of BV-2 cells

BV-2 cells were cultured in DMEM containing 10% FBS along with penicillin (50 U/mL) and streptomycin (50 μg/mL). Prior to transferring to culture plates, the FBS level was adjusted to 0.5%. A suspension containing 5 × 10^4^ cells/mL was distributed into 96-well plates (200 µL per well) and maintained in a humidified atmosphere with 5% CO_2_ at 37 °C.

#### 2.2.2 Culture and induced differentiation of N2A cells

N2A cells were maintained in MEM containing 10% FBS along with penicillin (50 units/mL) and streptomycin (50 μg/mL). Given their tumorigenic characteristics and insufficient expression of dopaminergic neuronal markers, these cells require differentiation induction for experimental applications. Following standard procedures, the serum concentration was progressively decreased to 0.5% FBS while supplementing with 1 mM db-cAMP to initiate cellular differentiation. The differentiation medium consisted of MEM with reduced serum (0.5% FBS), differentiation-inducing agents (1 mM db-cAMP), and standard antibiotic concentrations. Cellular suspensions were plated in 96-well culture dishes at 5 × 10^4^ cells/mL. Experimental treatments were administered after routine medium renewal at 48-h intervals.

### 2.3 Protective effect of iridoids on rotenone-induced N2A cells

**FIGURE 2:**
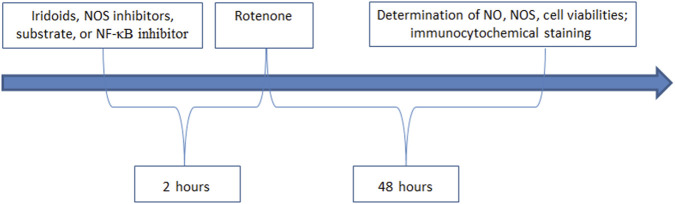
Experimental intervention and determination.

The study involved six experimental groups: control, rotenone-treated group, catalpol positive control, harpargide, acetylharpagide, and harpagoside. N2A cells were plated in 96-well plates at 5 × 10^4^ cells/mL density (200 µL/well) and cultured for 12 h. Following this initial culture period, the respective groups received 10 µM concentrations of harpargide, harpagoside, acetylharpagide, or catalpol. After a 2-h exposure period, all treatment groups were supplemented with 20 nM rotenone for subsequent 48-h cultivation (Figure 2). The rotenone-treated group exclusively received rotenone to induce cellular injury, while the control group remained untreated with both rotenone and iridoid derivatives. Cellular survival rates were quantitatively determined through CCK-8 assay measurements.

At specified intervals, 10 µL of CCK-8 solution was administered to individual wells followed by a 2-h maintenance period in the CO_2_ culture chamber. Optical density readings were subsequently obtained through microplate analysis using dual-wavelength detection (450 nm as primary detection wavelength and 620 nm as secondary reference wavelength). The entire procedure was conducted through three independent experimental replicates with the exception of the ELISA.

The cell viability percentage was determined using the equation: Survival rate = (As-Ab)/(Ac-Ab). In this formula, As corresponds to experimental wells containing cell culture medium, CCK-8 solution, and test compounds; Ac indicates control wells with medium and CCK-8 without test substances; Ab refers to blank wells comprising only medium and CCK-8 reagent without cellular components.

### 2.4 The nitric reductase method employed to measure the content of NO

#### 2.4.1 Experimental grouping

##### 2.4.1.1 Impact of iridoid compounds on NO levels in rotenone-treated cellular supernatants

The study comprised six experimental groups: a control group, a rotenone-treated group (20 nM), a catalpol reference group, along with harpagide, acetylharpagide, and harpagoside intervention groups, with the iridoid compounds administered at a final concentration of 10 µM in these four experimental conditions (Figure 2).

##### 2.4.1.2 Effect of harpagoside on NO content in rotenone-induced cell supernatant

The study comprised six experimental cohorts: the control group, rotenone-treated group (20 nM), catalpol-treated positive control group, and four harpagoside treatment arms with ascending concentrations (0.01, 0.1, 1, and 10 µM respectively).

##### 2.4.1.3 Impact of harpagoside on NO levels in cellular supernatants under NOS inhibition or enzymatic substrate (X) conditions

The experimental design comprised five distinct cohorts: a control group, a rotenone-treated group (20 nM), a harpagoside-treated group (10 µM concentration), an X group, and a combination X + harpagoside group. In this context, X denoted pharmacological agents including: the non-selective NOS inhibitor L-NMMA (NG-monomethyl-L-arginine monoacetate salt, administered at 1 mM), the iNOS-selective antagonist SMT (S-methylisothiourea sulfate, 1 mM concentration), the NOS enzymatic substrate L-arginine (1 mM), or the NF-κB pathway inhibitor BAY11-7082 (5 µM dosage).

#### 2.4.2 Content determination

##### 2.4.2.1 Standard curve preparation

The initial 1 mM standard solution was formulated by combining 5 µL from a 100 mM nitrate stock solution with 495 µL of detection buffer. Subsequently, aliquots of 0, 1, 2, 3, 4, and 5 µL from this working standard were added to individual reaction vessels, each supplemented with detection buffer to achieve a total volume of 42.5 µL, thereby generating a series of calibration samples.

##### 2.4.2.2 Reduction reaction

First, the blank well received an initial aliquot of 100 µL detection buffer. Standard and sample wells (excluding the blank) were then supplemented with 2.5 µL nitrate reductase mixture. This was followed by the addition of 2.5 µL enzyme cofactor to these same wells. The plate then underwent a 60-min room temperature incubation to facilitate nitrate-to-nitrite conversion. Finally, 2.5 µL enhancer solution was introduced, with a subsequent 10-min incubation period completing the process.

##### 2.4.2.3 Nitroso assay

Twenty-five microliter aliquots of Griess Reagent R1 and R2 were pipetted sequentially into both standard and experimental sample wells. The combined solutions underwent a 10-min ambient temperature incubation period, ensuring chromogenic reaction stability for up to 60 min. Spectrophotometric analysis was conducted at 540 nm wavelength to quantify optical density values.

### 2.5 The levels of iNOS determined by ELISA

#### 2.5.1 Experimental grouping

##### 2.5.1.1 Impact of harpagoside treatment on iNOS levels within rotenone-stimulated BV-2 microglial cell lysates

The study design incorporated five experimental groups: a control group, a rotenone-treated group, and three harpagoside-treated groups receiving 0.1, 1, and 10 µM concentrations respectively.

##### 2.5.1.2 Impact of harpagoside on iNOS levels within rotenone-stimulated BV-2 cellular homogenates under conditions of NF-κB inhibition with BAY11-7082

The experimental design comprised five distinct groups: control group, rotenone-treated group, harpagoside-treated groups (10 µM concentration), BAY11-7082 NF-κB inhibitor administration (5 µM dosage), and a combined treatment group receiving both BAY11-7082 (5 µM) and harpagoside (10 µM).

The sample content (n) corresponded to the number of replicate wells present on the culture plate at the time of the experiment.

#### 2.5.2 Content determination

Prior to initiating experimental procedures, all chemical solutions were acclimated to ambient laboratory conditions (maintained at 25 °C ± 2 °C rather than physiological temperature). During dilution processes, thorough mixing was performed cautiously to prevent bubble generation when preparing working concentrations. Preliminary assessments of sample concentrations were conducted through spectrophotometric analysis before formal measurements. For samples surpassing the kit’s detection limits, appropriate dilution adjustments were implemented with subsequent calculations incorporating the dilution coefficient to maintain quantitative accuracy. ①Sample preparation: Blank, standard, and experimental wells were set up. Each well received 100 µL of standard or test solution, excluding blank controls. After sealing with enzyme-conjugated covers, plates underwent 120-min incubation at 37 °C. During sample dispensing, particular attention was paid to direct liquid deposition at well bottoms, preventing air bubble formation and wall contact. Fresh standard solutions were routinely prepared prior to each assay to maintain procedural reliability. ② Following solution removal and gentle blot drying (without washing steps), 100 µL of freshly prepared Solution A (mixed within 30 min of use) was introduced to all wells. After thorough mixing, plates were maintained at 37 °C for 60 min ③ Post-incubation liquid was discarded and plates underwent triple washing cycles using 400 µL per well, with 1–2 min intervals between washes. Complete drying followed each washing phase through careful blotting. ④Subsequent addition of 100 µL Solution B preceded another 60-min incubation at 37 °C. Five identical washing cycles were then performed as described in the previous step. ⑤ Chromogenic development commenced with 90 µL substrate introduction per well, followed by dark-condition incubation at 37 °C. Color reaction timing was strictly maintained within a precise 15–30 min window, with initial wells typically displaying distinct blue gradients while terminal wells showed reduced chromatic variation. ⑥ Sequential addition of 50 µL termination solution induced immediate color transition from blue to yellow. Timely solution application upon reaction completion proved critical for measurement accuracy. ⑦ Immediate spectrophotometric analysis at 450 nm wavelength was conducted using microplate readers following termination, with optical density (OD) values recorded within 10 min of reaction cessation.

#### 2.5.3 Protein content determination

A volume of 25 µL of standard or sample was aspirated (linear range: 20–2000 μg/mL). A volume of 200 µL of Pierce® BCA protein quantification kit working solution was added, followed by thorough mixing for 30 s. The mixture was incubated at 37 °C for 30 min, cooled to room temperature, and the absorbance was measured at 562 nm.

### 2.6 Immunocytochemical staining

#### 2.6.1 Experimental grouping

N2A and BV-2 cells were cultured together in 96-well plates using a 1:4 cell ratio, with each well containing 200 µL of medium and a total cell density of 5 × 10^4^ cells/mL. The culture medium consisted of a DMEM:MEN mixture (1:4 ratio) containing 0.5% FBS, 0.1 mM db-cAMP, 50 U/mL penicillin, and 50 μg/mL streptomycin. Five experimental groups were established: a control group, a rotenone-treated group, and three harpagoside treatment groups with final concentrations of 0.1, 1, and 10 µM.

#### 2.6.2 Immunocytochemical staining procedure

① Specimens were taken out of refrigeration and left to reach ambient temperature. Each well underwent three 5-min PBS rinses using phosphate buffered saline. ② Specimens received treatment with 3% hydrogen peroxide solution (H_2_O_2_) under ambient conditions for 20 min to neutralize endogenous peroxidase. Three consecutive PBS washes lasting 5 min each followed this treatment. ③ Membrane permeability enhancement was achieved through 30-min exposure to 0.3% Triton X-100 at room temperature. This process was succeeded by three 5-min PBS cleaning cycles. ④ Blocking of non-specific interactions involved 30-min immersion in 5% bovine serum albumin (BSA) at ambient temperature. Subsequent PBS rinses were performed thrice with 5-min intervals. ⑤ Primary antibody application utilized mouse anti-human anti-nitration α-Syn (1:100 dilution) with overnight incubation at 4 °C. Post-refrigeration thermal conditioning occurred at 37 °C for 60 min. Negative controls utilized 5% BSA substitution for primary antibodies. Post-incubation cleansing involved triple PBS washes of 5-min duration. ⑥ Secondary anti-mouse/rabbit antibodies (1:1) were introduced for 60-min thermal incubation at 37 °C. Subsequent PBS rinsing was conducted three times with 5-min intervals. ⑦ SABC reagent application preceded 30-min ambient temperature incubation. Three sequential PBS washes (5 min each) followed this stage. ⑧ Chromogenic visualization employed DAB substrate under microscopic observation. Staining progression was halted immediately upon reaching optimal intensity, with digital image capture for permanent documentation. ⑨ At a magnification of ×200, 10 images from each group were captured using Eclipse TS100 inverted microscope (Nikon in every experiment. The number of positive cells in each image was quantified manually, and the average value was calculated to represent the cell count.

### 2.7 Statistical analysis

All experimental results are expressed as mean values with standard error (mean ± SEM) and underwent statistical processing through SAS software (version 6.12). ANOVA was employed for comparisons across multiple groups, with subsequent application of the SNK procedure for detailed pairwise analysis when significant variations emerged. For direct comparisons within paired experimental designs, paired Student *t*-tests were implemented as the analytical method. Before conducting ANOVA or student t-tests, the normality of data distribution and homogeneity of variance were evaluated. If the variables did not follow a normal distribution or exhibited heterogeneous variances, non-parametric statistical analysis was conducted using the Mann-Whitney U test. Throughout the study, probability values below 0.05 were established as the threshold for determining statistical significance.

## 3 Results

### 3.1 Protective effect of four iridoids on rotenone-induced N2A cells

**FIGURE 3:**
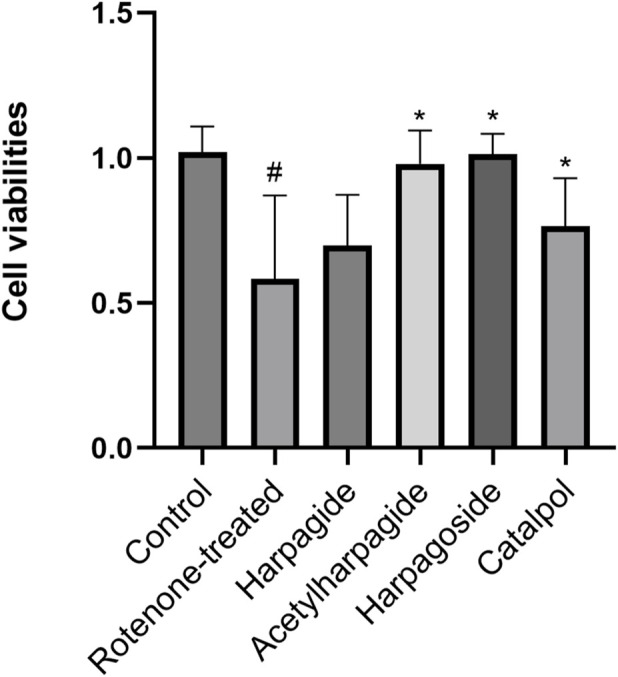
Protective effects of iridoids on N2A cell viabilities induced by rotenone. Cell viability of N2A cells exposed to 20 nM rotenone and pretreated with 10 µM iridoids (harpagide, harpagoside, acetylharpagide, catalpol) was assessed using CCK-8 assay. Data represent mean ± SEM of three independent experiments (n = 3). ANOVA was employed for comparisons across multiple groups, with subsequent application of the SNK procedure for detailed pairwise analysis when significant variations emerged. ^#^P < 0.05, versus control group; *P < 0.05, versus rotenone-treated group.

Figure 3 illustrates the neuroprotective properties of four iridoid compounds against rotenone-induced cytotoxicity in N2A cells. Following 48-h exposure to 20 nM rotenone, cellular viability decreased markedly from 1.020 ± 0.044 in control specimens to 0.583 ± 0.144 in rotenone-treated group, reflecting a 42.8% reduction in survival capacity (P < 0.05). Pre-administration of acetylharpagide, harpagoside, and catalpol effectively restored cell viability to 0.980 ± 0.057, 1.013 ± 0.035, and 0.764 ± 0.083 respectively, showing statistical significance (P < 0.05) in each case. Conversely, harpagide treatment yielded a survival rate of 0.697 ± 0.088, which failed to demonstrate a significant improvement relative to rotenone-treated group (P > 0.05).

### 3.2 Effect of iridoids on NO in rotenone-induced cells

#### 3.2.1 Effect of iridoids on NO levels in N2A Cells Induced by rotenone

**FIGURE 4:**
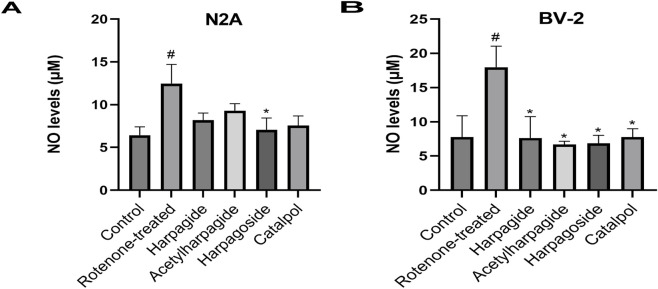
Effects of iridoids on NO levels in culture supernatants of N2A **(A)** or BV-2 cells **(B)**. Supernatant NO levels of N2A or BV-2 cells exposed to 20 nM rotenone and pretreated with 10 µM iridoids (harpagide, harpagoside, acetylharpagide, catalpol) was assessed using nitroso assay. Data represent mean ± SEM of three independent experiments (n = 3). ANOVA was employed for comparisons across multiple groups, with subsequent application of the SNK procedure for detailed pairwise analysis when significant variations emerged. ^#^P < 0.05, versus control group; *P < 0.05, versus rotenone-treated group.

As shown in Figure 4A, NO concentrations measured 6.44 ± 0.48 µM in control group supernatants. Following 48-h exposure to 20 nM rotenone, rotenone-treated group exhibited a 93.8% elevation (P < 0.05) in NO contents, reaching 12.48 ± 1.11 µM. Harpagoside pretreatment effectively reduced NO levels to 7.07 ± 0.69 µM (P < 0.05) compared with rotenone-treated group. No statistically significant alterations in NO concentrations were observed in groups pretreated with harpagide, acetylharpagide, or catalpol.

#### 3.2.2 Effect of iridoids on NO levels in BV-2 cels induced by rotenone

As shown in Figure 4B, NO concentration measured 7.79 ± 1.79 µM in control group supernatants, while rotenone-treated group exhibited a 130.6% elevation (17.96 ± 1.79 µM) after 48-h exposure (P < 0.05). However, pre-administration of harpagide, acetylharpagide, harpagoside and catalpol for 2 h prior to rotenone exposure significantly lowered NO concentrations to 7.63 ± 1.82, 6.68 ± 0.28, 6.84 ± 0.69, and 7.79 ± 0.69 µM respectively (P < 0.05 versus rotenone-treated group). Comparative analysis revealed no statistically meaningful variations in NO measurements among the four iridoid pretreatment groups (P > 0.05).

### 3.3 Effect of harpagoside on NO levels in rotenone-exposed N2A and BV-2 cellular supernatants

#### 3.3.1 Effect of harpagoside on NO content in the supernatant of N2A Cells Induced by rotenone

**FIGURE 5:**
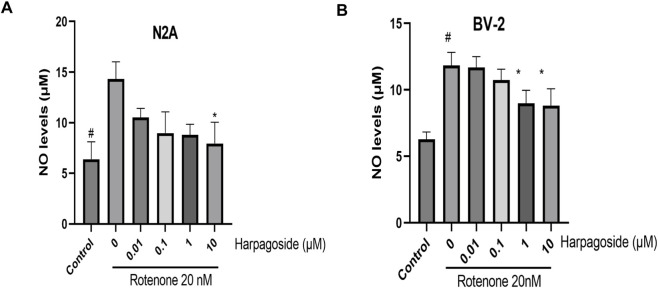
Effects of harpagoside on NO content in the supernatant of N2A **(A)** and BV-2 cells **(B)**. Supernatant NO levels of N2A or BV-2 cells exposed to 20 nM rotenone and pretreated with harpagoside (0.01, 0.1, 1, and 10 µM) was assessed using nitroso assay. Data represent mean ± SEM of three independent experiments (n = 3). ANOVA was employed for comparisons across multiple groups, with subsequent application of the SNK procedure for detailed pairwise analysis when significant variations emerged. ^#^P < 0.05, as compared with control group; *P < 0.05, as compared with rotenone-treated group.

Figure 5A demonstrates that baseline NO concentrations in control group supernatants measured 6.39 ± 0.86 µM. Following 48-h exposure to 20 nM rotenone, rotenone-treated group exhibited a significant rise in NO levels to 14.31 ± 0.86 µM (123.9% elevation, P < 0.05). Pre-administration of 10 µM harpagoside for 2 h effectively lowered NO concentrations from rotenone-treated group values to 8.80 ± 0.52 µM, representing a 38.5% reduction (P < 0.05). Lower harpagoside concentrations (0.01–1 µM) failed to produce significant alterations in supernatant NO levels compared to rotenone-treated group.

#### 3.3.2 Effect of harpagoside on rotenone-induced NO content in BV-2 cell supernatant

Figure 5B demonstrates that control group supernatants exhibited NO concentrations of 6.28 ± 0.32 µM. Following 48-h exposure to 20 nM rotenone, rotenone-treated group showed a significant rise in NO levels to 11.84 ± 0.57 µM, representing an 88.5% increase compared to controls (P < 0.05). Two-hour pretreatment with 1 and 10 µM harpagoside effectively lowered NO concentrations to 8.98 ± 0.57 µM and 8.82 ± 0.73 µM respectively in rotenone-treated groups, demonstrating statistically significant reductions (both P < 0.05). In contrast, administration of 0.01 and 0.1 µM harpagoside did not produce statistically significant alterations in supernatant NO levels.

### 3.4 Effect of harpagoside on iNOS levels in BV-2 cell homogenates induced by rotenone

**FIGURE 6:**
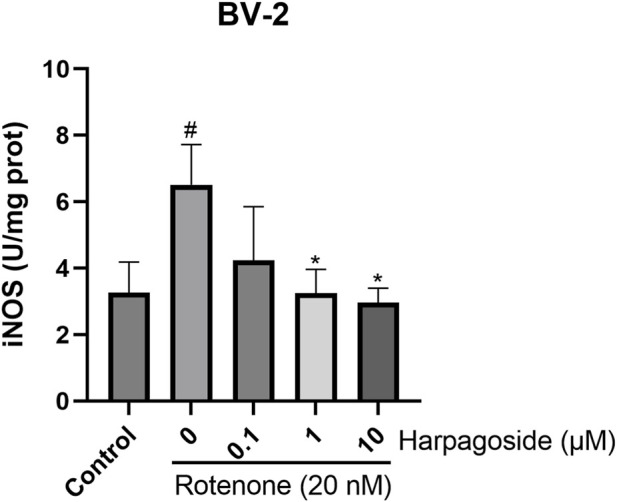
Effects of harpagoside on iNOS levels in homogenates of BV-2 cells. iNOS levels of BV-2 cells exposed to 20 nM rotenone and pretreated with harpagoside (0.1, 1, and 10 µM) was assessed using ELISA. Data represent mean ± SEM of 3 replicate wells on the cultivation plate (n = 3). ANOVA was employed for comparisons across multiple groups, with subsequent application of the SNK procedure for detailed pairwise analysis when significant variations emerged. *P < 0.05, as compared with rotenone-treated group.

As illustrated in Figure 6, the iNOS levels in the control group was measured at 3.27 ± 0.53 U/mg protein. In contrast, the rotenone-treated group exhibited a significantly elevated iNOS levels of 6.51 ± 0.70 U/mg protein, representing a 99.1% increase compared to the control group (P < 0.05). Pretreatment with harpagoside at concentrations of 1, 10 µM effectively reduced the iNOS levels to 3.25 ± 0.41 and 2.96 ± 0.25 U/mg protein, respectively, corresponding to reductions of 50.1% and 54.5% (P < 0.05 for both). However, pre-administration of 0.1 µM harpagoside did not result in a statistically significant change in iNOS levels (4.24 ± 0.93 U/mg protein vs. 6.51 ± 0.70 U/mg protein, P > 0.05).

### 3.5 Effect of harpagoside on NO content in rotenone-induced N2A and BV-2 cells in the presence of NOS inhibitors

#### 3.5.1 Effect of harpagoside on NO content in the rotenone-induced N2A cells in the presence of NOS inhibitors

**FIGURE 7:**
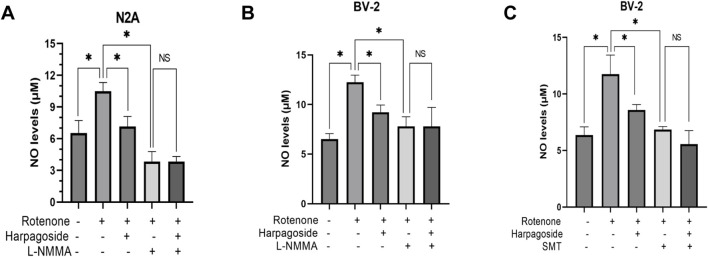
Effects of harpagoside with or without L-NMMA or SMT on NO levels in culture supernatants of N2A **(A)** or BV-2 cells **(B,C)**. Supernatant NO levels of N2A or BV-2 cells exposed to 20 nM rotenone and pretreated with harpagoside (10 µM) with or without L-NMMA (1 mM) or SMT (1 mM) was assessed using nitroso assay. Data represent mean ± SEM of three independent experiments (n = 3). ANOVA was employed for comparisons across multiple groups, with subsequent application of the SNK procedure for detailed pairwise analysis when significant variations emerged. *P < 0.05, ^NS^P > 0.05.

As illustrated in Figure 7A, baseline NO levels measured 6.52 ± 0.69 µM in control specimens, whereas rotenone-exposed samples exhibited elevated concentrations of 10.49 ± 0.48 µM, demonstrating a 60.9% elevation relative to controls (P < 0.05). Harpagoside pretreatment effectively attenuated this increase, reducing rotenone-treated group NO values from 10.49 ± 0.48 µM to 7.15 ± 0.55 µM (31.8% decrease, P < 0.05). Co-administration with L-NMMA (NOS inhibitor) further diminished NO levels to 3.82 ± 0.55 µM in the rotenone-treated group (P < 0.05). Crucially, pre-administration harpagoside concurrent with L-NMMA 2 h before rotenone exposure showed no statistically meaningful impact on NO concentrations (3.82 ± 0.55 vs. 3.82 ± 0.28 µM, P > 0.05).

#### 3.5.2 Effect of harpagoside on NO content in the rotenone-induced BV-2 cells in the presence of NOS inhibitors

As shown in Figure 7B, control group supernatant exhibited NO concentrations of 6.52 ± 0.32 µM, while rotenone-exposed samples demonstrated a marked increase to 12.24 ± 0.42 µM, representing an 87.7% surge compared to controls (P < 0.05). When harpagoside was administered prior to rotenone treatment, NO concentrations were reduced by 24.7% (9.22 ± 0.42 µM) relative to the rotenone-treated group (P < 0.05). Following treatment with the NOS inhibitor L-NMMA, rotenone-treated group NO concentrations dropped significantly to 7.79 ± 0.57 µM (P < 0.05). Notably, pre-administration with harpagoside and L-NMMA prior to rotenone exposure did not produce a statistically significant alteration in NO levels (7.79 ± 0.57 vs. 7.79 ± 1.11 µM, P > 0.05).

As shown in Figure 7C, control group supernatant exhibited NO concentrations measuring 6.36 ± 0.42 µM, whereas rotenone exposure elevated this value to 11.76 ± 0.97 µM, demonstrating a significant 84.9% elevation relative to controls (P < 0.05). Pre-treatment with harpagoside effectively lowered NO concentrations from rotenone-treated group’s 11.76 ± 0.97 µM to 8.58 ± 0.28 µM, indicating a notable 27.0% decline compared to rotenone-treated group (P < 0.05). Following SMT intervention, rotenone-treated group NO levels decreased substantially to 6.84 ± 0.16 µM (P < 0.05). Notably, harpagoside administration showed no statistically significant impact on NO concentrations when administered with SMT 2 h before rotenone exposure (5.56 ± 0.69 µM vs. 6.84 ± 0.16 µM, P > 0.05).

### 3.6 Effect of harpagoside on NO content in rotenone-induced N2A, BV-2 cells in the presence of NOS substrate arginine

#### 3.6.1 Effect of harpagoside on NO content in the rotenone-induced N2A cells in the presence of the NOS substrate arginine

**FIGURE 8:**
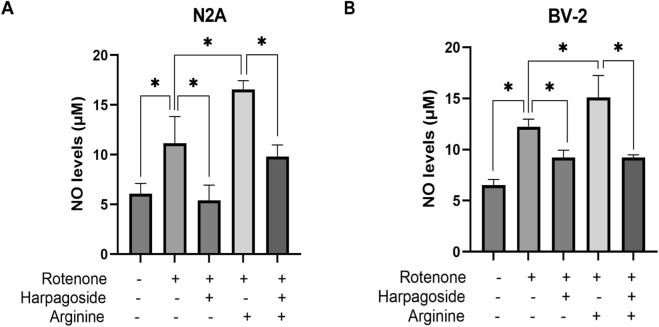
Effects of harpagoside with or without arginine on NO levels in culture supernatants of N2A **(A)** or BV-2 cells **(B)**. Supernatant NO levels of N2A or BV-2 cells exposed to 20 nM rotenone and pretreated with harpagoside (10 µM) with or without arginine (1 mM) was assessed using nitroso assay. Data represent mean ± SEM of three independent experiments (n = 3). ANOVA was employed for comparisons across multiple groups, with subsequent application of the SNK procedure for detailed pairwise analysis when significant variations emerged. *P < 0.05.

As shown in Figure 8A, control group supernatant exhibited NO concentration of 6.08 ± 0.59 µM, while rotenone-treated samples demonstrated significantly elevated NO levels at 11.15 ± 1.55 µM, representing an 83.4% increase compared to controls (P < 0.05). Harpagoside pretreatment effectively decreased NO concentrations from rotenone-treated group’s 11.15 ± 1.55 µM to 5.40 ± 0.89 µM, achieving a 51.6% reduction (P < 0.05). Pre-administration of arginine (NOS substrate) notably increased NO levels in rotenone-treated group to 16.55 ± 0.89 µM (P < 0.05). Notably, harpagoside intervention during combined rotenone-arginine exposure successfully counteracted this increase, maintaining NO levels at 9.80 ± 0.68 µM (P < 0.05).

#### 3.6.2 Effect of harpagoside on NO content in the rotenone-induced BV-2 cells in the presence of the NOS substrate arginine

As depicted in Figure 8B, the control group displayed a nitric oxide concentration of 6.52 ± 0.32 µM in supernatant, while the rotenone-treated group exhibited significantly elevated NO levels at 12.24 ± 0.42 µM, representing an 87.7% increase compared to controls (P < 0.05). Harpagoside pre-administration substantially reduced NO content from rotenone-treated group’s 12.24 ± 0.42 µM to 9.22 ± 0.42 µM, demonstrating a 24.7% decrease (P < 0.05). Following arginine (NOS substrate) administration, NO levels in rotenone-treated group rose to 15.10 ± 1.24 µM (P < 0.05). Notably, harpagoside treatment effectively counteracted this elevation under combined rotenone-arginine exposure, lowering NO concentration to 9.22 ± 0.16 µM (P < 0.05).

### 3.7 Effect of harpagoside on NO content in N2A and BV-2 cells induced by rotenone in the presence of NF-κB inhibitor BAY11-7082

#### 3.7.1 Effect of harpagoside on NO content in rotenone-induced N2A cells in the presence of NF-κB inhibitor BAY11-7082

**FIGURE 9:**
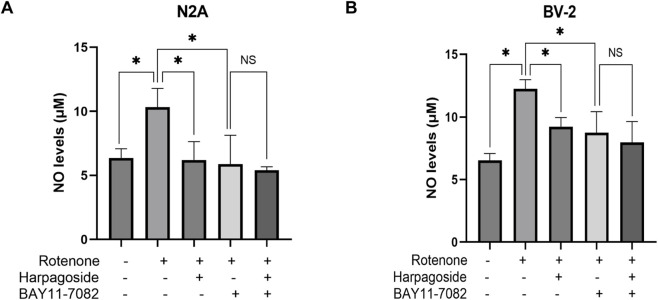
Effects of harpagoside with or without BAY11-7082 on NO levels in culture supernatants of N2A **(A)** or BV-2 cells **(B)**. Supernatant NO levels BV-2 cells exposed to 20 nM rotenone and pretreated with harpagoside (10 µM) with or without BAY11-7082 (5 µM) was assessed using nitroso assay. Data represent mean ± SEM of 3 replicate wells on the cultivation plate (n = 3). ANOVA was employed for comparisons across multiple groups, with subsequent application of the SNK procedure for detailed pairwise analysis when significant variations emerged. *P < 0.05, ^NS^P > 0.05.

As shown in Figure 9A, control group supernatant exhibited NO concentrations of 6.36 ± 0.42 µM, while the rotenone-treated group demonstrated a 62.4% elevation (10.33 ± 0.84 µM) compared to controls (P < 0.05). Harpagoside pre-administration significantly reduced NO levels in rotenone-treated group by 39.8%, decreasing from 10.33 ± 0.84 µM to 6.20 ± 0.83 µM (P < 0.05). Pretreatment with NF-κB inhibitor BAY11-7082 further lowered NO concentrations to 5.88 ± 1.30 µM in rotenone-treated group (P < 0.05). Notably, when co-administered with both rotenone and BAY11-7082, harpagoside showed no statistically significant impact on nitric oxide levels (5.40 ± 0.16 µM vs. 5.88 ± 1.30 µM, P > 0.05).

#### 3.7.2 Effect of harpagoside on NO in the rotenone-induced BV-2 cells in the presence of NF-κB inhibitor BAY11-7082

As illustrated in Figure 9B, NO concentrations measured 6.52 ± 0.32 μM in control group supernatants, while the rotenone-treated group demonstrated a marked elevation to 12.24 ± 0.42 μM, representing an 87.7% increase compared to controls (P < 0.05). Following harpagoside pre-administration, NO levels in rotenone-treated group showed significant attenuation, decreasing by 24.7% from 12.24 ± 0.42 μM to 9.22 ± 0.42 μM (P < 0.05). The introduction of NF-κB inhibitor BAY11-7082 further reduced NO concentrations in rotenone-treated group to 8.74 ± 0.97 μM (P < 0.05). Notably, when both rotenone and BAY11-7082 were present, harpagoside treatment exhibited no statistically significant effect on NO levels (8.74 ± 0.97 μM vs. 7.95 ± 0.97 μM, P > 0.05).

### 3.8 Effect of harpagoside on iNOS in a BV-2 cells induced by rotenone in the presence of the NF-κB inhibitor BAY11-7082

**FIGURE 10:**
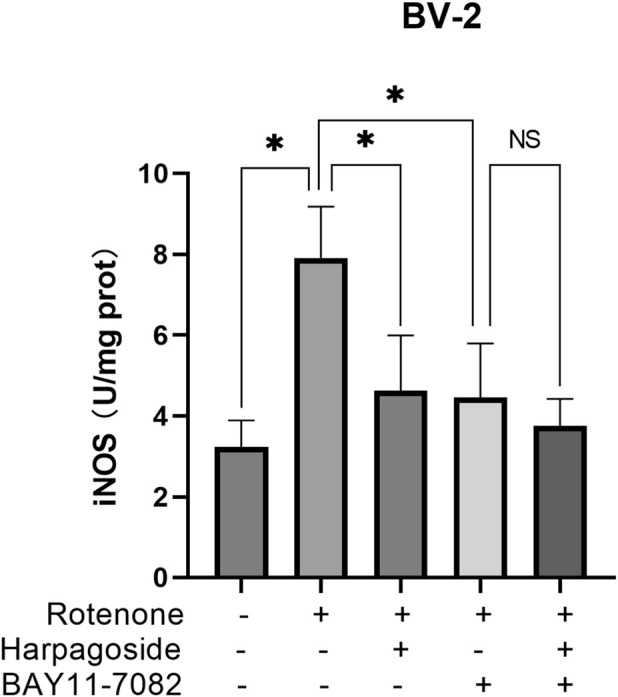
Effects of harpagoside with or without BAY11-7082 on iNOS levels in homogenates of BV-2 cells. iNOS levels of BV-2 cells exposed to 20 nM rotenone and pretreated with harpagoside (10 µM) with or without BAY11-7082 (5 µM) was assessed using ELISA. Data represent mean ± SEM of three independent experiments (n = 3). ANOVA was employed for comparisons across multiple groups, with subsequent application of the SNK procedure for detailed pairwise analysis when significant variations emerged. *P < 0.05, ^NS^P > 0.05.

As shown in Figure 10, the control group exhibited iNOS levels of 3.23 ± 0.38 U/mg protein, while the rotenone-treated group demonstrated a 144.9% elevation (7.91 ± 0.73 U/mg protein) compared to controls (P < 0.05). Following intervention, rotenone-treated group’s iNOS concentration decreased by 41.6% from baseline values (7.91 ± 0.73 to 4.62 ± 0.79 U/mg protein, P < 0.05). Pre-administration of the NF-κB inhibitor BAY11-7082 further reduced iNOS levels to 4.46 ± 0.77 U/mg protein in rotenone-treated group (P < 0.05). Notably, harpagoside treatment showed no significant effect on iNOS levels when co-administered with rotenone and BAY11-7082 (4.46 ± 0.77 vs. 3.75 ± 0.39 U/mg protein, P > 0.05).

### 3.9 Effects of harpagoside on rotenone-induced nitration of α-syn in N2A/BV-2 Co-culture

**FIGURE 11:**
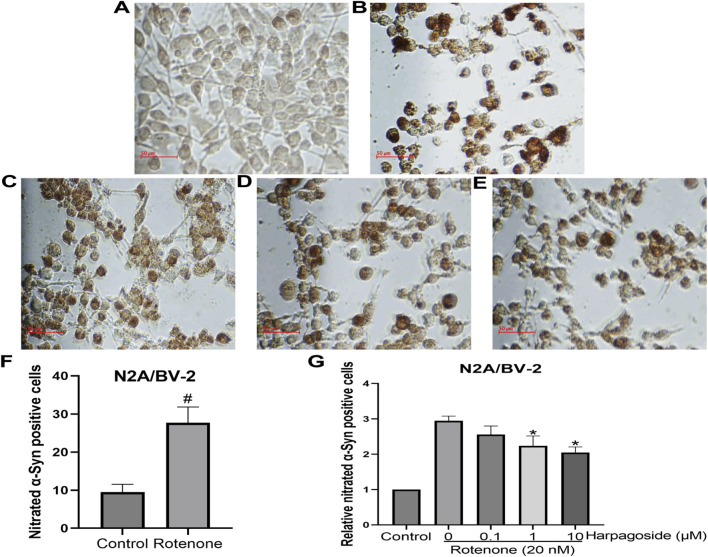
Effects of harpagoside on nitrated α-Syn positive cells in the cultures of N2A/BV-2 cells **(A)** Conrol group; **(B)** Rotenone-treated group; **(C)** Rotenone+0.1 μM Harpagoside; **(D)** Rotenone+1 μM Harpagoside; **(E)** Rotenone+10 μM Harpagoside. Student *t*-test was used for comparison between control and rotenone-treated group **(F)**. Then nitrated α-Syn positive cell count of the control group is established as 100%, and the cell counts of the other groups are standardized in relation to this benchmark **(G)**. Nitrated α-Syn positive cells in N2A/BV-2 co-culture exposed to 20 nM rotenone and pretreated with harpagoside (0.1, 1, 10 µM) were assessed using immunocytochemical staining. Data represent mean ± SEM of three independent experiments (n = 3). Student’s t-test was used for comparison between control and rotenone-treated group. ANOVA was employed for comparisons across multiple groups, with subsequent application of the SNK procedure for detailed pairwise analysis when significant variations emerged. ^#^P < 0.05, as compared with control group, *P < 0.05, as compared with rotenone-treated group.

As shown in Figure 11, untreated controls exhibited 9.52 ± 1.17 nitrated α-Syn-positive cells, while rotenone-exposed samples demonstrated significantly elevated counts of 27.74 ± 2.38 (P < 0.05), representing a 1.95-fold elevation compared to untreated controls. Following standardized intervention, rotenone-treated specimens displayed reduced nitrated α-Syn levels at 2.95 ± 0.13. This rotenone-induced elevation was further mitigated by harpagoside pre-administration, with 1 μM and 10 µM concentrations decreasing values to 2.24 ± 0.16 and 2.05 ± 0.09 respectively (both P < 0.05). Notably, the 0.1 µM harpagoside concentration showed no significant alteration in nitrated α-Syn levels (2.56 ± 0.24 vs. rotenone group’s 2.95 ± 0.13, P > 0.05).

## 4 Discussion

α-Syn is a crucial pathogenic protein implicated in PD, positioning it as a primary therapeutic target for disease modification (Hassanzadeh et al., 2024). Aggregates formed through the overexpression of wild-type α-Syn do not exhibit toxicity towards human neuronal cells (Ko et al., 2008). Increased phosphorylation and nitration of α-Syn have been observed in the substantia nigra of aging primates (McCormack et al., 2012). Furthermore, Enhanced nitration of α-Syn and an associated neuroinflammatory response were observed in aged rats (Choi et al., 2010). Microglial inflammation affects the rate of nigrostriatal degeneration in PD, partly triggered by misfolded, nitrated α-synuclein (N-α-Syn) from Lewy bodies released by dying dopaminergic neurons (Reynolds et al., 2009). Neuroinflammation and α- Syn dysfunction exacerbate each other, accelerating chronic neurodegeneration in a mouse model of PD (Gao et al., 2011). Selective nitration of α-Syn in PD links oxidative and nitrative damage to the onset and progression of neurodegenerative synucleinopathies (Giasson et al., 2000). Targeting RNS may provide therapeutic benefits for individuals in the early clinical stages of PD (Stykel and Ryan, 2022). In this investigation, we demonstrated the protective effects of iridoids, including acetylharpagide, harpagoside, and catalpol, on rotenone-intoxicated N2A cells *in vitro.* Subsequently, our focus shifted to elucidating the role of the NF-κB/NOS/NO/α-Syn nitration signaling pathway in mediating the protective effect of harpagoside, which was selected as the representative compound for further investigation.

N2A cells are extensively utilized to investigate neuronal differentiation, axonal growth, and signaling pathways. These cells express Nurr-related factor 1 while exhibiting limited expression of tyrosine hydroxylase (TH) and low levels of dopamine. The application of N2A cells without differentiation induction has been documented in various neurological studies (Yurchenko et al., 2020; Lee et al., 2020; Tao et al., 2024; Kang et al., 2017; Xia et al., 2023; Menchinskaya et al., 2021). To address the differences between the N2A cell line and primary DA neurons, we employed a differentiation induction protocol to promote the transformation of N2A cells into DA neuron-like cells. Several research groups have implemented strategies to promote cell differentiation by reducing the concentration of FBS in the culture medium while additionally introducing various differentiation-inducing agents. N2A cells were induced to differentiate using retinoic acid (RA) or allowed to differentiate over a period of 48 h in the absence of FBS (Namsi et al., 2018). In another differentiation study, the culture medium was switched to DMEM with 0.5% FBS after overnight growth and then cultured for an additional 3 days with 1 mM db-cAMP (Bajpai et al., 2013). The introduction of db-cAMP resulted in a significant enhancement of TH expression and an increase in dopamine levels, as demonstrated by Western blot analysis, immunocytochemistry, and high-performance liquid chromatography (Tremblay et al., 2010). Earlier work by our team established that 10 μmol/L harpagoside improved cellular viability in db-cAMP differentiated neuronal cultures challenged with rotenone (Lang and Xiong, 2025). Subsequent stdy by our research group documented harpagoside’s ability to attenuate tyrosine hydroxylase (TH)-positive neuronal depletion and counteract axonal retraction in 1-methyl-4-phenylpyridimium (MPP^+^)-treated mesencephalic neuron cultures (Sun et al., 2012). The compound also dose-dependently preserved nigral TH-positive neuronal populations in chronic MPTP mouse models while enhancing motor coordination, as quantified through rotarod performance assessments (Sun et al., 2012).

Plant-derived iridoids, such as catalpol and harpagoside, have been shown to exhibit significant neuroprotective effects and possess the ability to slow down the process of neurodegeneration in PD (Dinda et al., 2019). Catalpol, harpagide, harpagoside, and acetoharbacoside share a common parent nucleus structure, which is classified as an iridoid. In the investigation of the protective effects of iridoids in an *in vitro* PD cell model, catalpol, harpagoside, and acetoharbacoside demonstrated significant efficacy against rotenone-induced N2A cells, The dosage of iridoids was determined based on the previous research findings of our research group. Catalpol at concentrations of 1 × 10^−5^ M and 1 × 10^−4^ M demonstrates protective effects on the number of TH-positive neurons as well as their neurite outgrowth length in rat mesencephalic cultures intoxicated with MPP^+^ (Xu et al., 2010). Harpagoside at concentrations of 1–10 μM protects dopaminergic neurons in rat midbrain cultures against MPP^+^-induced toxicity *in vitro*, while concentrations ranging from 10 to 100 μM can effectively reverse the damage (Sun et al., 2012). Harpagoside at concentrations ranging from 0.1 to 10 μM effectively alleviated rotenone-induced mitochondrial swelling in N2A cells (Lang and Xiong, 2025).

The antioxidant and anti-inflammatory effects of harpagoside have been documented in the scientific literature as follows. It has been reported that oxidative damage induced by fluphenazine is characterized by alterations in catalase activity and levels of ROS within the cortex and striatum. This oxidative stress was significantly alleviated by the ethyl acetate fraction of *Harpagophytum procumbens*, particularly in the striatum (Schaffer et al., 2016). Treatment with E-harpagoside in scopolamine-induced amnesic mice significantly reduced thiobarbituric acid reactive substance levels and enhanced the activities or concentrations of glutathione reductase, superoxide dismutase (SOD), and reduced glutathione (GSH). Therefore, E-harpagoside exhibited notable cognitive-enhancing effects primarily through its antioxidant mechanisms (J et al., 2008). Harpagoside, a prominent iridoid glycoside found in devil’s claw, has been identified as one of the active components in *Harpagophytum procumbens* extract (Inaba et al., 2010). It was observed to inhibit the production of interleukin-1β, interleukin −6, and tumor necrosis factor-α in RAW 264.7 cells (Inaba et al., 2010). The fraction with the highest concentration of harpagoside exhibited a non-selective inhibition of COX-1 and COX-2 activities, resulting in reductions of 37.2% and 29.5%, respectively (Anauate et al., 2010). Additionally, it significantly inhibited NO production by 66% using a whole blood assay (Anauate et al., 2010) and IL-6 expression in primary human osteoarthritis chondrocytes (Haseeb et al., 2017).

Experimental findings reveal that *Harpagophytum procumbens* alleviates arsenic-induced neurobehavioral modifications and neuronal toxicity in female rodents. The therapeutic potency showed dose-dependent correlation with circulating harpagoside concentrations, confirming this compound as the principal neuroprotective agent (Peruru et al., 2020). Scientific investigations demonstrate that *Harpagophytum procumbens* extracts elevate brain-derived neurotrophic factor (BDNF) gene expression in amyloid β-peptide-treated rat cortical synaptosomal preparations (Ferrante et al., 2017). Mechanistic studies reveal harpagoside counteracts amyloid-β-mediated cognitive dysfunction through dual modulation of BDNF synthesis and concurrent stimulation of mitogen-activated protein kinase (MAPK)/phosphatidylinositol 3-kinase (PI3K) signaling networks (Li et al., 2015). The compound also demonstrates efficacy against MPTP/MPP^+^-triggered dopaminergic pathway degeneration and motor deficits via GDNF potentiation (Sun et al., 2012). Notably, harpagoside preserves mitochondrial bioenergetics in rotenone-modeled Parkinsonian cellular systems (Lang and Xiong, 2025).

The most harpagoside-enriched fraction suppressed NO production by 66% in whole-blood assays (Anauate et al., 2010). Purified harpagoside demonstrated concentration-dependent inhibition (0.3–1 mg/mL) of iNOS in rat renal mesangial cells (Kaszkin et al., 2004). Mechanistic studies identified NF-κB pathway suppression as the mechanism behind harpagoside’s inhibition of lipopolysaccharide-induced iNOS expression in HepG2 and RAW 264.7 cell lines (Huang et al., 2006). In hyperuricemic murine models, harpagoside treatment attenuated inflammatory cell infiltration and modulated NF-κB expression, mitigating renal damage (Fu et al., 2024).

In this investigation, rotenone exposure was employed to establish PD pathology models using N2A and BV-2 cell lines. Previous research demonstrates that subacute administration of this pesticide induces motor dysfunction and reduces TH-positive neurons in the SN of rats, potentially correlated with increased cerebral NO concentrations (Xiong et al., 2015). The gaseous signaling molecule NO, principally generated through NOS-catalyzed L-arginine oxidation (Gu and Zhu, 2020), exerts dual roles in neuroinflammatory regulation. Within PD pathogenesis, it amplifies central nervous system (CNS) inflammatory cascades while maintaining crucial defensive functions against pathogenic agents at physiological concentrations (Subedi et al., 2021; Martins-Pinge et al., 2022). Pathological overproduction of this reactive nitrogen species initiates oxidative stress-mediated cellular apoptosis and perpetuates inflammatory signaling pathways (Subedi et al., 2021). Emerging research highlights NO metabolic imbalance, particularly its excessive synthesis, as a potential catalyst in neurological disease progression. Substantial evidence implicates NO dysregulation in prevalent neurodegenerative disorders including PD (Iova et al., 2023). During neuroinflammatory responses, activated glial cells (microglia and astrocytes) generate substantial NO quantities (Liy et al., 2021), with microglial-derived NO overproduction being specifically associated with PD-related neurodegeneration and chronic inflammation (Subedi et al., 2021).

The findings of this investigation revealed that harpagoside displayed notable neuroprotective properties within the rotenone-exposed N2A cellular model. As a characteristic iridoid constituent isolated from *Scrophulariae*, harpagoside underwent comprehensive analysis regarding its biological activities. Experimental data revealed its substantial impact on NO generation, with marked elevation in NO concentrations detected within culture supernatants from both N2A and BV-2 cellular systems. This NO upregulation was mechanistically associated with harpagoside’s modulation of rotenone-induced iNOS expression in BV-2 microglial models. Pathological overproduction of NO, particularly through iNOS and neuronal nitric oxide synthase (nNOS) pathways, has been extensively correlated with neuroinflammatory responses and oxidative/nitrosative stress mechanisms that accelerate neuronal degeneration in PD pathogenesis (Tripodi et al., 2025). Clinical imaging studies utilizing [18F]NOS tracers documented significantly expanded cerebral distribution volumes in PD patients relative to age-controlled healthy subjects (Doot et al., 2022). Experimental models employing 6-hydroxydopamine-induced striatal lesions demonstrated N-methyl-D-aspartate receptor reorganization triggering nNOS activation (de Carvalho et al., 2024). Excessive nNOS-derived NO facilitates detrimental post-translational protein modifications that disrupt cellular biochemistry and compromise CNS functionality, with nNOS dysregulation being a recognized feature in PD progression (Maccallini and Amoroso, 2023). Protein analysis techniques including Western blotting and immunohistochemistry have identified upregulated iNOS expression within the substantia nigra pars compacta (SNpc) following MPTP administration (Ren et al., 2015). Genetic association studies revealed significant correlations between PD susceptibility and polymorphisms in eNOS (intron 4 VNTR), iNOS (exon 22 A/G), and nNOS (exon 29 T/C) isoforms (Huerta et al., 2007). Particularly strong associations emerged for iNOS exon 22 and nNOS exon 29 variants, while nNOS exon 18 polymorphisms showed no statistically significant correlation (Levecque et al., 2003). This study investigates the effects of harpagoside on the expression of nNOS and iNOS, utilizing N2A cells and BV-2 cells as *in vitro* models to represent dopamine neurons and microglia, respectively. This approach is based on the understanding that primary rat mesencephalic culture systems comprise not only DA neurons but also various other neuronal cell types and microglia.

The experimental findings indicated that treatment with rotenone elevated the quantity of nitrated α-Syn-positive cells within the N2A/BV-2 co-culture model. Notably, administration of harpagoside prior to rotenone exposure markedly reduced α-Syn nitration levels. PD is pathologically defined by the progressive loss of dopaminergic neurons and abnormal accumulation of aggregated α-Syn, particularly within the SNpc region (Martirosyan et al., 2024; Henderson et al., 2019). Cellular diversity within the SN, encompassing DA neurons and other cell populations, contributes to the intricate molecular alterations underlying PD development (Wang et al., 2024). The nitration of tyrosine residues and the formation of covalent α-Syn dimers are notable examples observed in post-mortem brain sections from individuals with PD (Schildknecht et al., 2013). Peroxynitrite (ONOO^−^) is recognized as one of the most potent biological nitrosative agents, generated at exceptionally rapid rates when NO and superoxide (•O_2_^-^) are combined (De Simone et al., 2025). The gradual infusion of pre-formed peroxynitrite into mixtures containing α-Syn and HCO_3_^−^ significantly enhanced both the nitration and aggregation of α-Syn, primarily through the formation of dityrosine (Andrekopoulos et al., 2004). The nitrated α-Syn has the potential to induce cell death in a manner that is both time- and concentration-dependent (Liu et al., 2011).

The limitations of this study were as follows: the constraints associated with the *in vitro* rotenone model, the reliance on immortalized cell lines rather than primary dopaminergic neurons or glial cells, and the lack of genetic approaches (e.g., siRNA) and *in vivo* validation. Subsequently, our research team will advance to the next phases of investigation, which include genetic approaches and *in vivo* animal models, with the aim of translating these findings into clinical significance.

In summary, the protective effects of harpagoside on cellular models exposed to rotenone were achieved by regulating the NF-κB/NOS/NO/α-Syn nitration signaling cascade. Furthermore, in another study, we observed protective effects of harpagoside on mitochondrial function in rotenone-induced cellular models of PD (Lang and Xiong, 2025). However, the potential relationship between the effects of harpagoside on NOS and NO levels, as well as the subsequent nitration of α-Syn and its protective role in mitochondrial function, warrants further investigation in future studies. To a certain extent, the findings may illuminate future *in vivo* or clinical investigations, while concurrently elucidating the potential significance of harpagoside within the broader context of disease modification for PD.

## Data Availability

The raw data supporting the conclusions of this article will be made available by the authors, without undue reservation.
